# A patent-based analysis of the evolution of basic, mission-oriented, and applied research in European universities

**DOI:** 10.1007/s10961-023-10001-5

**Published:** 2023-03-22

**Authors:** Gabriele Angori, Chiara Marzocchi, Laura Ramaciotti, Ugo Rizzo

**Affiliations:** 1 Department of Economics and Management, University of Ferrara, Ferrara, Italy; 2 Newcastle University Business School, Newcastle University, Newcastle, England, UK; 3 Department of Mathematics and Computer Sciences, University of Ferrara, Ferrara, Italy; 4 SEEDS, Sustainability, Environmental Economics and Dynamics Studies, Ferrara, Italy

**Keywords:** University research, Industry research, Patents, Division of labour, Use-inspired basic research, I23, O33

## Abstract

The dynamics of basic and applied research at university and industry have steadily changed since the Eighties, with the private sector reducing its investments in science and universities experiencing significant remodelling in the governance of their funding. While studies have focussed on documenting these changes in industry, less attention has been paid to observe the trajectories of basic and applied research in universities. This work contributes to fill this gap by looking at the evolution of publicly funded research that has been patented by universities between 1978 and 2015. First, we adopt a critical perspective of the basic versus applied dichotomy and identify patents according to three typologies of research: basic, mission-oriented, and applied research. Second, we describe the evolution of these three typologies in universities compared to industry. Our results show that over the years, patents from academic research that was publicly funded have become more oriented towards pure basic research, with mission-oriented basic research and pure applied research decreasing from the late 1990s. These results complement and extend the literature on basic and applied research dynamics in the private sector. By introducing mission-oriented research as a type of basic research with consideration of use, the work problematises the basic and applied research dichotomy and provides insights into the evolution of academic research focus, offering a more complex picture of how university research contributes to industry and broader social value creation.

## Introduction

The relevance of university–industry interactions and their effects on economic growth and innovation have been undisputed for many years. Positive returns from R&D collaboration are spurred by technology transfer, workforce up-skilling, or the leveraging of public–private R&D complementarities in technology enhancement and diffusion. A recent example is the successful joint venture between the Jenner Institute at Oxford University and the private company AstraZeneca, which collaborated at unprecedented speed to create a COVID-19 vaccine. While the response to the call for vaccine development was swift (Astrazeneca: April 2020^1^) and successful (Oxford University: November 2020^2^; Corey et al., 2020), it also exposed some acrimonious debates about the contribution of public versus private funding to the process of discovery (Cross et al., 2021), and the IPRs management issues between the parts involved (Nature, 2021). Discussions about COVID-19 vaccine development chime well with recent literature revealing the tensions in university–industry collaboration, and the public perceptions surrounding the value for society that publicly funded university research generates (Druedahl et al., 2021; Fink et al., 2022; Kovac & Rakovec, 2022; Kyle, 2022).

Since the 1980s, universities have remodelled the governance of public funding and institutionalised third mission activities. Changes to the rationale of research funding occurred in response to two dynamics. First, government funding has become clustered across fewer institutions (Jongbloed & Lepori, 2015), and while it remains the main source of research income for universities, third-party funding has increased in relative terms (Geuna, 2001; Lepori et al., 2007). Second, governance and attribution mechanisms of public funding have moved from core-funding (block grants) to competitive allocation systems (Cocos & Lepori, 2020; Hicks, 2012; Lepori et al., 2007). Some countries, such as the UK, also started to accrue funding based on research impact, i.e.: the outlined contribution of research to social value and national priorities (Watermeyer, 2016). While increasing competition for resources, such allocation frameworks tend to support short-term projects focussing on broad top-down priorities (Lepori, 2011) and have channelled criticisms for their contractual-oriented mode (Geuna, 2001). These appraisals, labelled marketization of resource allocation (Jongbloed & Lepori, 2015), sportification of science (Kaldewey, 2018), and academic capitalism (Much, 2014) mostly converge to reflect the concern that higher education policy has been remodelled as a subset of economic policies, negatively affecting academic freedom in terms of research (Rhoades and Slaughter 1997).

A second relevant change impacting university research occurred with the consolidation of third mission and commercialisation activities, placing the contribution to economic development at the forefront of universities’ missions. Third-mission activities have embedded economic exploitation into academic research: first, they aimed to increase research monetisation in a context of decreasing government spending, and second, they promoted the transfer of academic research otherwise left on the shelf. Throughout the 1990s and 2000s, third mission activities became mainstream, and many universities established bespoke infrastructures (such as TTOs) and implemented incentives to encourage academic staff to exploit research in the market (e.g. Baldini et al., 2014; Lockett et al., 2015). On one side, engagement activities have brought benefits for the scientists involved (e.g. Gulbrandsen & Smeby, 2005; Haeussler & Colyvas, 2011), and have been considered in a positive light by governments and the broader society (e.g. Flink & Kaldewey, 2018; Gulbrandsen & Slipersaeter, 2007; Muscio et al., 2017). On the other hand, it has been noted that engagement with industry affects academics’ choice (Cooper, 2009), creating tensions particularly for those academics involved in basic research (Davies et al., 2011).

Despite from different perspectives, these discussions reflect concerns about the division of labour between university and industry and its impact in terms of value for society (e.g. Geuna & Martin, 2003; Geuna & Rossi, 2011; Rosenberg & Nelson, 1994). The division of labour between university and industry describes the balance between basic and applied research produced in the two sectors (Mowery & Sampat, 2004; Nelson & Romer, 1996). Literature shows that in recent years it has become more fragmented with companies moving away from basic research to focus on the applied and developmental phases of R&D (Arora et al., 2018). As emerged from the Covid experience, while ‘there remains a perception among some in industry that universities produce the science and leave industry to commercialize it’ (Nature, 2021, p. 302), industry’s strategy of disinvesting in basic research and sourcing science directly from universities has proved challenging (Arora et al., 2020). Issues concerning the division of labour between university and industry have been investigated from the perspective of the private sector, with only few empirical studies analysing their effects on the content of research carried out by universities (Li et al., 2017). In this paper, we look at this gap and investigate the evolution of basic and applied research in European universities. We build a sample using patents to proxy university research that has been publicly funded, and adopt a critical perspective on the dichotomy basic-applied by building on Stokes’ (1997) work (e.g. David & Hall, 2000; Sampat, 2012; Tijssen, 2018). By employing patent characteristics, we observe the evolution of the research content, i.e.: the focus of research activities carried out at universities, and unpack them into basic, mission-oriented and applied. Then, we look at the evolution of these three typologies comparing universities and industry filed patents. Our results show that in comparison to industry, applied and mission-oriented research content in university-owned patents stemming from government funding has declined and their basic research focus has increased. While patent analyses do not cover the full extent of the academic research output, our results bare suggestions consistent with recent literature adopting a broader range of indicators and expanding the analysis to include academic outputs such as publications (Park et al., 2023).

We provide two main contributions to the literature. First, we classify patent indicators into basic, mission-oriented and applied research by comparing three groups: industry patents, university patents and patents transferred from university to industry. Differently from the extant literature (e.g.: Czarnitzki et al., 2009; Trajtenberg et al., 1997), we use the latter group to empirically disentangle basic research into basic research with and without consideration of use (Stokes, 1997) and overcome the basic versus applied dichotomy. Second, we complement and extend existing literature focussing on the evolution of industry’s basic and applied research (Arora et al., 2018, 2020) by: first, expanding the analysis to mission-oriented research and hence providing a more nuanced understanding of basic research with consideration of use, and second, offering a novel picture on how basic, applied and mission-oriented research have evolved across European universities in the last forty years vis-à-vis industry.

The paper is structured as follows. Section 2 reviews the main literature, discussing the basic-versus-applied research dichotomy in terms of value creation (2.1), avenues to overcome such dichotomy (2.2), and the literature adopting patent indicators to study university research (2.3). Section 3 presents the sample and data employed in the analysis. Section 4 discusses the methods, including how patent indicators are sorted into basic, mission-oriented and applied, while Sect. 5 focuses on the empirical results concerning the evolution of the research focus of universities compared to industry. Sections 6 and 7 outline discussion and conclusions respectively.

## Background literature

### The social value generated by basic and applied research

Literature investigating the content of university research, i.e.: the focus of research activities carried out by universities, has adopted the basic versus applied dichotomy to challenge the underestimation of social value generated by academic work (Azoulay et al., 2009; Cockburn & Henderson, 1997; Li et al., 2017). Basic research is broadly considered abstract, ‘blue sky’, and curiosity driven. It involves research in pure science more exploratory than problem-solving and it’s perceived to carry little short-term value for society. On the other side, applied research is identified with practical problem-solving and it is regarded closer to exploitation by means of industrial development and more likely to create social value in the short term. However, adopting basic and applied as categories to assess the value of research is problematic, because it meddles with a comprehensive identification of the contribution of university research to society (Stokes, 1997; Fortunato et al., 2018).

In the past, university and industry sustained a balanced division of labour between basic and applied research (Rosenberg & Nelson, 1994). Basic research was considered mostly a domain of universities which benefitted by block-grants and full-time personnel with the freedom to develop risky and long-term research projects (Kline, 1995) industry could not commit to. Universities also had academic independence from business interests (Nelson & Romer, 1996), and the autonomy to pursue research trajectories translating into social impact only in the longer term. This is evidenced by the rich literature on ‘sleeping beauties’: academic discoveries left dormant because premature for scientific and industrial applications in their time (Stent, 1972; Van Raan, 2004), but later exploited and some awarded Nobel prize status (Li & Ye, 2012).

Over the last thirty years, changes to funding rationales and the creation of third-mission streams have raised concerns about governments shifting resources towards applied research, and in turn affecting the balance between basic and applied research activities by universities (Thursby & Thursby, 2011). Third-mission funding streams have skewed scientists’ intention to pursue basic research (Davies et al., 2011), which became of comparatively low relevance in modern universities (Nowotny et al., 2003). Also, collaboration with industry naturally shifted the research content of some disciplinary domains in the direction of applied research (Quaglione et al., 2015). Given research commercialization has been associated to lower data production and results dissemination in the academic community (Campbell et al., 2002), a stronger focus on applied research might restrict the capacity of university research to generate social value in the long term.

On the other hand, literature has investigated the returns to public investment and social value generated by basic research. Using U.S. National Institutes of Health (NIH) grant data, Li et al. (2017) find that basic research outputs are as likely to have an economic impact as applied research. This is particularly true where basic research produces technological progress (Comroe & Dripps, 1976; Macilwain, 2010; Moses et al., 2005), with scientists in disciplines traditionally considered science-focussed such as the life sciences and, to some extent physics, increasingly finding inspiration for their work in societal needs (Cohen et al., 2020). Hu (2020) shows that persistent funding in long-term basic research has led to increased collaboration activities such as publications (also in less research-oriented universities) and international networks. In a similar vein, Ahmadpoor and Jones (2017) show that 79.7% of the science and engineering literature can ultimately be linked to one patent. Altogether, this suggests that breakthrough research outcomes are more likely to emerge when research funding is based on long-term and experimental rewards (basic science) rather than on short-term, less explorative objectives (Azoulay et al., 2011).

While literature has focussed on identifying the impacts of third-mission activities and changes to the governance of funding on scientific productivity, an understanding of whether universities shifted the content of their research activities from basic to applied is scant (e.g., Azoulay et al., 2009; Li et al., 2017). Evidence suggests the division of labour in terms of research has evolved according to the knowledge demands of the business sector and the funding rationales for university research (Marburger III, 2005; Schauz, 2014). This also emerges from analyses looking at applied versus basic research expenditures dynamics in industry during the last fifty years (Arora et al., 2018, 2019; Zahra et al., 2018). In the inter-war period U.S. companies developed internal units devoted to basic science to compensate for the weakness of academic research in emerging sectors (Arora et al., 2021). Conversely, in recent years the business sector has been decreasing its investments in basic research (Arora et al., 2018, 2020). Between 1953 and 2017, the share of basic research carried out by U.S. companies has fallen from 32 to 26%,^3^ hinting that industry is drawing on academic basic research to compensate for the decline in corporate science (Arora et al., 2020; Bikard & Marx, 2020).

The literature above suggests that the value of basic research produced by universities is hard to tackle and broader than pure science without consideration of use (Marburger III, 2005). Research might be basic in nature and distant from commercial exploitation, but it could be stirred by a mission to solve well defined problems of application (Nelson & Romer, 1996). Indeed, some basic research develops on a continuum with applied research, particularly where it is motivated by finding resolutions to practical problems (Azoulay & Li, 2020; Nelson, 1959) and its value should be identified in relation to this translational capacity (Calvert, 2006).

### Consideration of use in university research: basic, applied and mission-oriented research

The dichotomisation of basic versus applied research is problematic: it lacks nuancing and overlooks the contribution of research motivated by a scientific quest but driven by the desire to find solutions of use to societal practical problems. To interpret research along the basic-applied continuum, Stokes (1997) reframes the understanding of basic and applied research using the concepts of ‘consideration of use’ and ‘quest for fundamental understanding’ and identifies three categories. The first is pure basic research, driven by a quest for fundamental understanding and no consideration of use. This research is involved with fundamental science and similar to the work of scientists such as Niels Bohr, who were motivated by a quest for ‘new to the world knowledge’ but not inspired by societal challenges. The second is use-inspired basic research, later in the literature defined as mission-oriented basic research (Nelson & Romer, 1996; Rosenberg & Nelson, 1994), which is driven by both the quest for fundamental understanding and consideration of use. This research resembles the work by scientists such as Pasteur: motivated by scientific curiosity but inspired in their quest by real-world problems. The third category is pure applied research stemming from research activities primarily concerned with consideration of use. Such approach is driven by a problem-solving attitude concerned by addressing market needs resembling the work of scientists such as Edison.

In Stokes’ taxonomy basic research is hybrid: it can be concerned with practical applications and in this format (Pasteur) link university and industry research to create an efficient division of labour between the two (Rosenberg & Nelson, 1994). Stokes approach to the classification of research typologies has been variously adopted in the literature to overcome the inefficiencies of the basic-applied dichotomy (Tijssen, 2018; Martínez et al., 2013; Akcigit et al, 2021). Ooms et al. (2015), show evidence that mapping regional specialisation with basic, applied and mission-oriented research activities provide a more realistic identification of local industrial patterns. Stokes framework has been found useful to investigate scientists’ own perception of their research. For instance, Bentley et al. (2015) by surveying over 12,000 scientists found that the majority combines basic and applied research in their scientific activities. Similar results are proposed by Gulbrandsen & Kyvik (2010) reporting that Norwegian scientists have difficulties in separating research in terms of basic or applied, and rather identify their research work as basic, applied research and a combination of both. Conversely, Amara et al. (2019) find that the majority of Spanish researchers are motivated by a quest for fundamental understanding (a-la Bohr), rather than by the consideration of use of their research (a-la Pasteur).

### Patent information as proxy of research focus

A number of studies have compared the characteristics of university-owned patents with the characteristics of company-owned patents (Czarnitzki et al., 2009; Sampat et al., 2003; Sapsalis et al., 2006; Sterzi et al., 2019; Trajtenberg et al., 1997). Many of these do so by looking at the content of basic research and associating it with specific features of patents. In this section, we review the indicators adopted to discuss the degree of basicness in research output and clarify how such indicators relate to pure basic, mission-oriented, and pure applied research.

In one of the first and most influential works using patents to disentangle the notion of basic research, Trajtenberg et al. (1997) define basicness in relation to fundamental features of innovation such as closeness to science and breadth. The paper first identifies a variety of measures to proxy these features and then tests how such features score in university and corporate patents. In particular, the authors assume that compared to firms, university patents will be cited with more intensity (higher number of forward citations) and across different sectors (higher generality index). The underlying rationale proposed by Trajtenberg et al. (1997) is that basicness shows proximity to science, that is, research undertaken by universities rather than corporations. Accordingly, university patents should display more references to scientific literature because of the proximity of the inventions to science (Callaert et al., 2006, 2014), but should also have a higher originality index, meaning that they would source references across a greater variety of technological fields. Following this seminal work, several studies have adopted forward citations (Henderson et al., 1998; Sampat et al., 2003; Sterzi, 2013; Sterzi et al., 2019; Thursby et al., 2009), generality index (Henderson et al., 1998; Mowery & Ziedonis, 2002; Sterzi, 2013), originality index (Sterzi, 2013; Thursby et al., 2009), and references to non-patent literature (Sapsalis and van Pottelsberghe de la Potterie, 2007; Rizzo et al., 2020) to study university patents.

Forward citations are considered a proxy for value and diffusion, and they are commonly used to study patent features and dynamics. Nonetheless, studies comparing university and company patents using forward citations have obtained mixed results (Lissoni & Montobbio, 2015), with some authors identifying a citation premium for university patents (e.g. Sampat et al., 2003; Trajtenberg et al., 1997), some finding no statistical difference (e.g. Sapsalis et al., 2006; Thursby et al., 2009), and others finding a citation premium for company patents (e.g. Czarnitzki et al, 2012; Sterzi, 2013). Other studies have looked at forward citations and distinguished long and short-term dynamics. If university patents are more likely to protect basic research, which should on average be more distant from direct commercial applications, university patents will receive the bulk of citations in the long term and relatively few in the short term. According to results from Czarnitzki et al. (2012), corporate patents where inventors are academic scientists tend to be cited more than university-owned patents within the first five years after filing; conversely, citations received more than five years after filing are greater for university-owned patents. Looking at the U.K., Sterzi (2013) and Sterzi et al. (2019) corroborate this with similar results.

Given the basicness of their content, on average university patent citations tend to increase later than corporate patents. Accordingly, the literature has also adopted citation time lag as a measure of research basicness (Czarnitzki et al., 2011; Sampat et al., 2003). In a similar vein, starting from the premise that basic research is further away from application compared to industrial applied research, patent oppositions can be considered an indication of basicness: Czarnitzki et al. (2009) provide evidence that university patents experience lower rates of opposition.

The indicators discussed so far have been applied to studying university patents and, sometimes, also to proxy the basicness of research content. This work also adopts additional indicators to explore the characteristics of university patents, although these have not been directly associated with the basicness of research content. For instance, patent family size is generally employed to measure the value of university and non-university patents, with a larger patent family size representing greater value (Sterzi et al., 2019) or quality (Cerulli et al., 2021). Another common indicator in the literature is patent scope, which has been adopted to measure complexity (Barbieri et al., 2020) as it represents the distinct technological components within an invention. Shane (2001) explores the factors increasing the probability of a patent to being brought to the market via firm creation, and finds that patent scope representing the most important determinant of such output. Finally, novelty is another indicator used to study university patents (Rizzo et al., 2020), given that scientific research is considered to lead to breakthrough inventions with a higher probability than company R&D (e.g. Thursby et al., 2009).

While the extant patent literature often adopts these indicators as a measure of the basicness of research, we move a step forward and associate them to basic, mission-oriented and applied levels of research. Specifically, we identify and compare industry patents, university patents, and patents transferred from university to industry and use the latter to disentangle basic research with and without destination of use.

## Sample and descriptive statistics

**Fig. 1:**
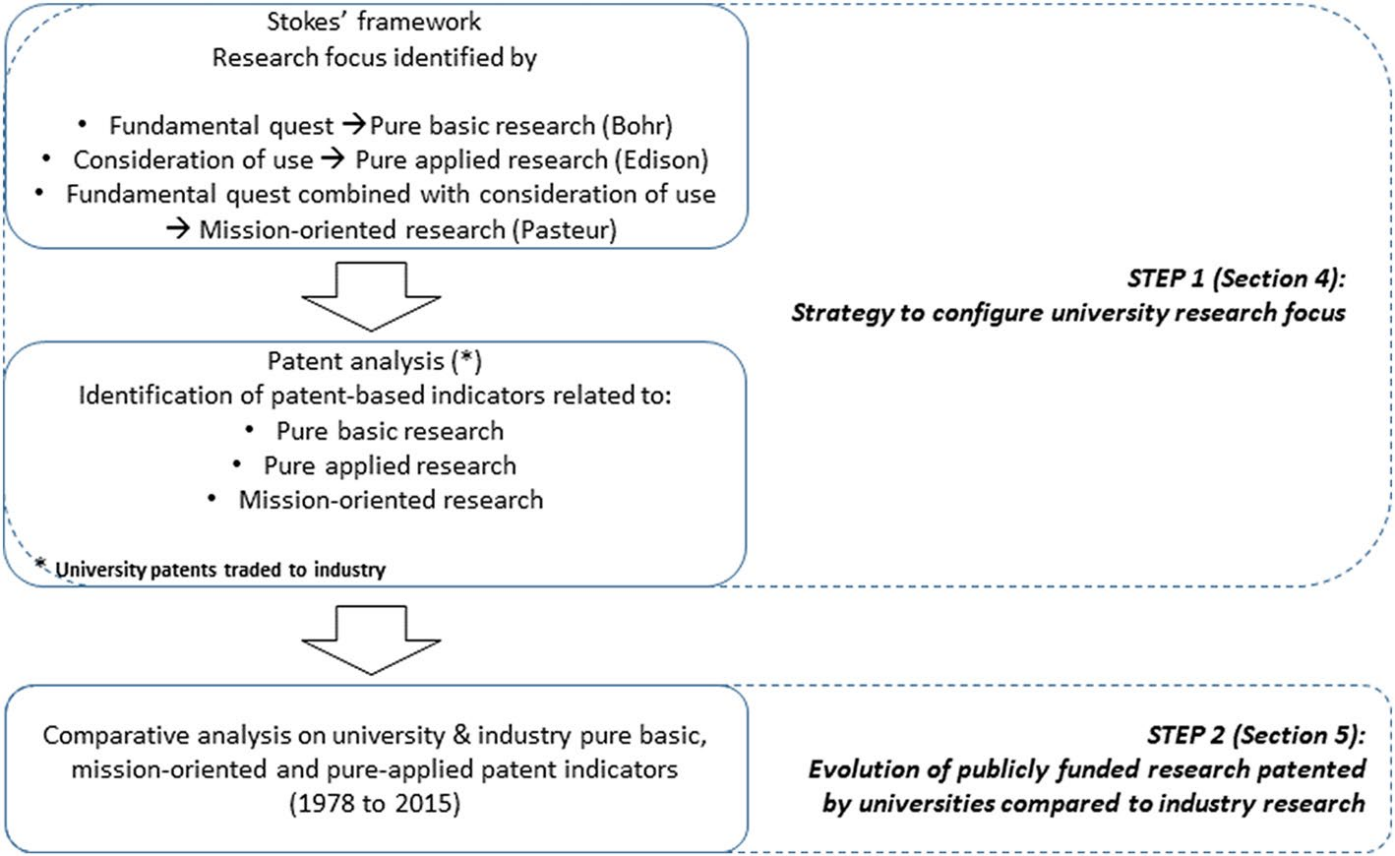
Structure of the paper: research strategy and objectives

The final aim of this work is to describe the evolution in content of publicly-funded research activities carried out at universities. We do so by exploiting patent information and exploring changes in the characteristics of university-owned patents^4^compared to company-owned ones.^5^In order to compare the evolution in the focus of publicly-funded university research, we first identify indicators to proxy the knowledge content within patents to associate patent-based indicators to Stokes’ quadrants (1997). Once identified indicators proxying basic research, mission-oriented research, and applied research, we observe the evolution of these indicators in university patents compared to industry patents. Figure 1 summarises these passages, and illustrates schematically how the paper is structured, from the theoretical premises to the final contribution.

This work builds on patent data to study university research evolution. It is known that patents do not allow a precise characterisation of academic research activity and for this reason our results must be taken with caution. Despite the limits involved in adopting patent data to proxy for university research, patents have the advantage to provide a wealth of information allowing a detailed characterisation of research content. In the next sections, we first describe the source of data and present some descriptive statistics on university and industry patents (Sect. 3). Following, we present in detail the methodologies adopted to classify patents indicators as proxy of different research focuses—basic, mission oriented, and applied—(Sect. 4), and map the evolution of basic, mission oriented and applied research in universities from 1978 to 2015 (Sect. 5).

### Data

We rely on information retrieved from the Worldwide Patent Statistical Database (Patstat), and more specifically from two distinct sources: Patstat Global and the Patstat Register (Spring Edition, 2019). The former includes a variety of bibliographic information on patents, and the latter contains procedural and legal-status information on patent applications that allows the tracking of ownership changes. Modifications to the legal status are recorded in the database when a transfer (or other administrative event) occurs that produces changes to the ownership structure.

From Patstat Global, we first select European Patent Office (EPO) patents and identify applicants based in Europe. Second, we select all patents with either public or private ownership, thus excluding patents where the applicant is an individual, a non-profit organisation, or unknown. We then distinguish private ownership from university, government, or other public ownership, and select only patents that are either only-privately owned or only-publicly owned. In so doing, building on the assumption that all patents emerging from a collaboration with the private sector will be either owned by both the university and the firm, or only by the firm, we determine a sample with only patents stemming from publicly funded research.

We focus on identifying changes in the ownership structure (through Patstat Register) and identify university-owned patents transferred to private companies (Orsatti & Sterzi, 2018). The analysis of this group’s characteristics will later be used to sort the contents of the (patented) university research output into pure basic, mission-oriented, and applied research, that is, to map university research content to Stokes’ quadrants. In order to avoid double counting across patents filed in more than one country, we adopt the patent family as the unit of analysis (Hall & Helmers, 2013). However, this does not guarantee identical claims and disclosure conditions since patent filing procedures can vary among patent offices and patent issuing authorities (Simmons, 2009). In order to cope with multiple equivalents, we follow an established approach and select the highest value within the family for each variable used in the analysis^6^ (e.g. Barbieri et al., 2020; Verhoeven et al., 2016).

From the starting population of EPO patents whose applicants’ addresses correspond to European countries, we restrict the sample to those countries where public research organisations own at least 1,000 patents filed in the 1978–2015 period.^7^ The countries satisfying this threshold are the U.K., Germany, Switzerland, France, Italy, the Netherlands, Spain, and Belgium. The sample of countries selected does not differ from other works based on European academic patents (e.g. Lissoni & Montobbio, 2015; Martínez & Sterzi, 2021). The sample consists of 1.1 million patents, of which 27 thousand are university-owned (2.5%). We then exclude university–industry co-owned patents (4103 patents), patent families with missing information (206 patents, corresponding to 0.2%), and patents with extreme values (about 60 patents). The final sample comprises 1,111,834 patent families with 22,920 university-owned patents. Among these, 2914 have been transferred from a university to the private sector, that is, 12.7% of university-owned patents changed ownership from public to private during the time period considered.

### Industry and university patenting activity (1978–2015)

**Fig. 2:**
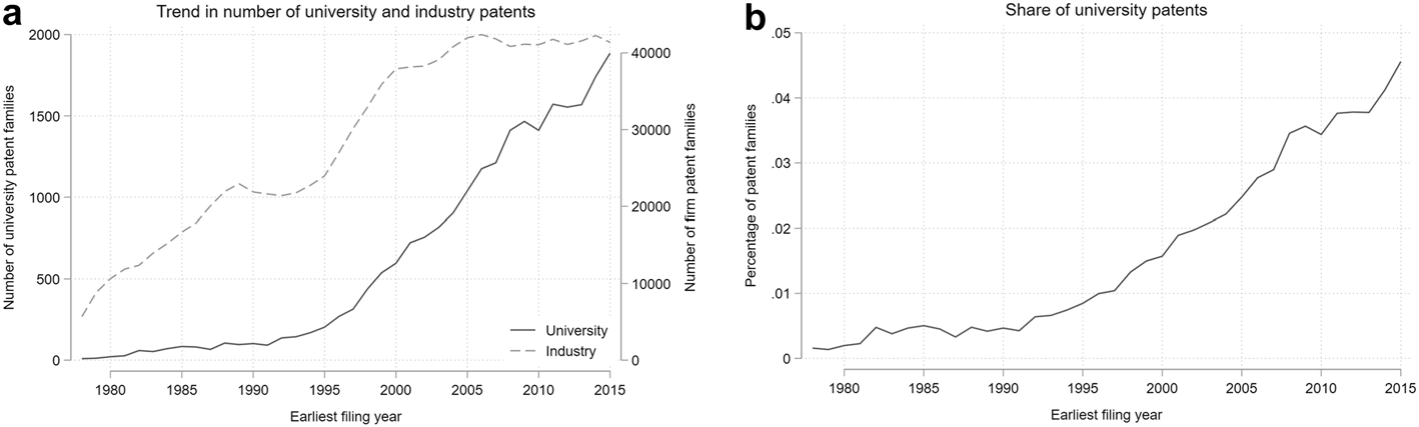
Number of university and industry patent family applications (left/panel a) and share of university patents on industry patents (right/panel b) by earliest filing year

This section presents descriptive analyses of university patenting activity and its evolution within the timeframe considered. Figure 2 reports the yearly number of university-owned patent families (panel a) and the share they represent out of all patents (panel b). The number of patents filed by universities increases slowly from 1978 to the early 1990s and then increases both more rapidly and steadily, while industry’s rate of growth is more pronounced in the 1980s and 1990s and then stabilises from 2000 onwards.^8^ Finally, the right-hand figure shows that the share of university patents out of all patents increases steadily across the whole timeframe analysed.

**Fig. 3:**
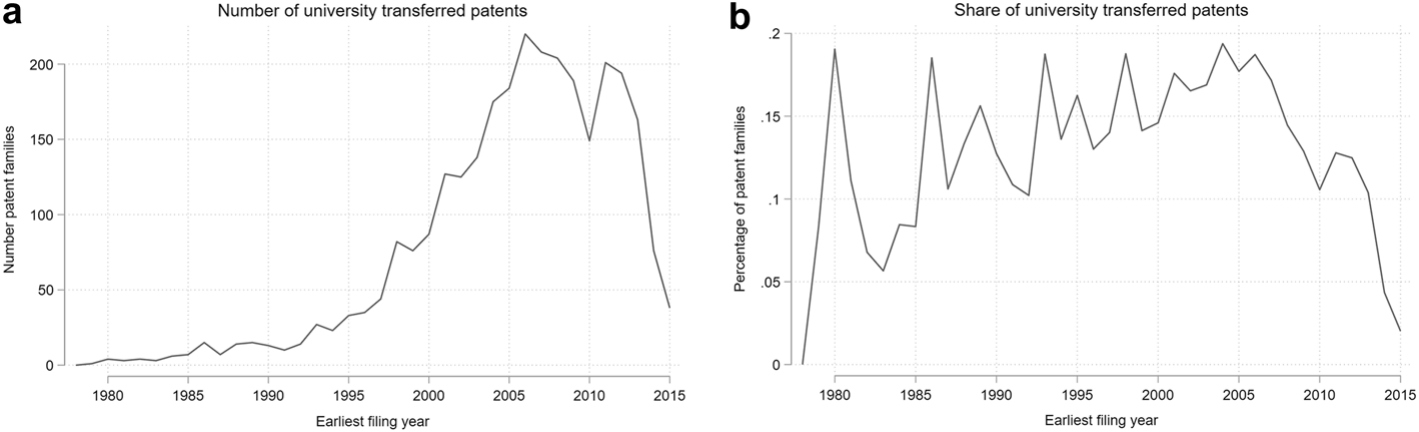
Number (left/panel a) and share (right/panel b) of university patents transferred to industry

Figure 3 describes the trend in the share of university patents transferred to industry. We can see from panel (a) that the number of traded university patents first takes off in the mid-1990s and then decreases after 2005. While the final decrease in patents might suffer from a truncation issue (as some patent transfers might have yet to occur), the tipping point at which patents begin to reduce is not subject to this problem. Therefore, we can confirm that the number of transferred patents has reduced since the mid-2000s. On the right-hand side (panel b), we observe that the share of transferred patents on the total number of university patents slowly increased up to the mid-2000s and then started to decrease. Truncation issues (as above) still apply, but the initial decrease in share reported here does not suffer from it.

## Identification of basic, mission-oriented and applied research focus in patents

**Fig. 4:**
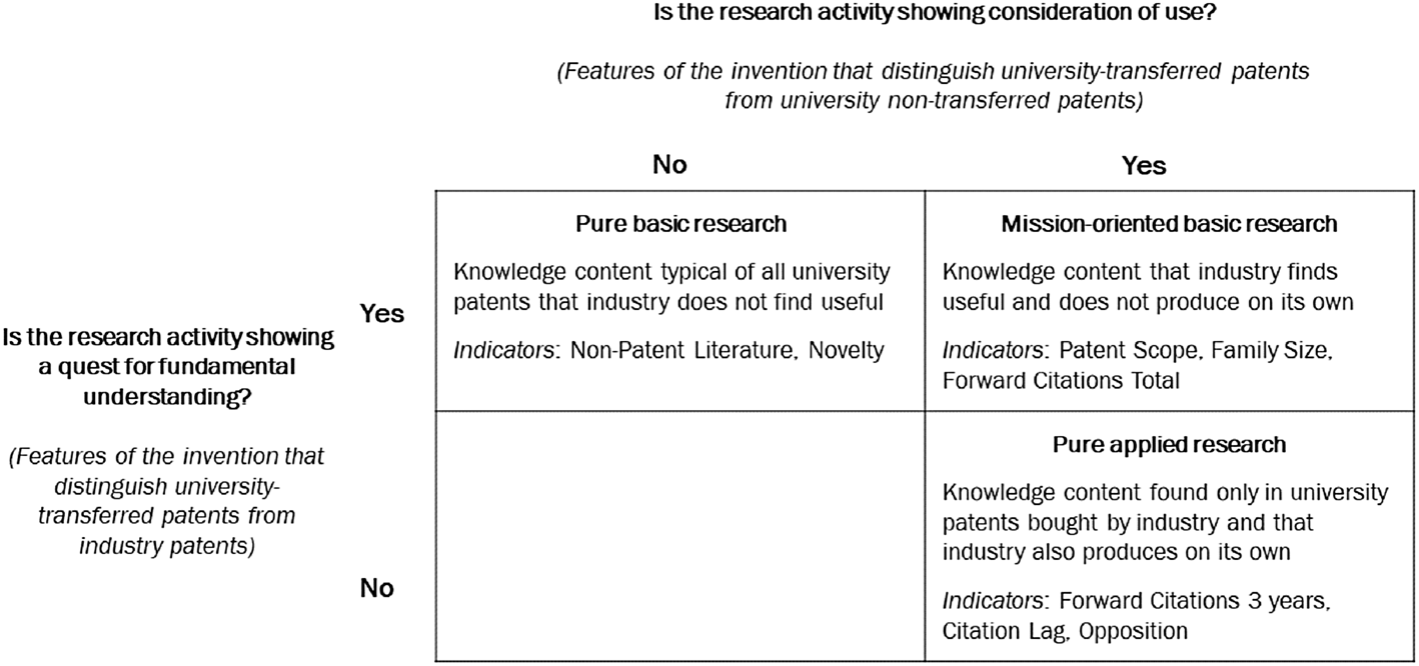
Adapted Stokes quadrants framework and corresponding patent indicators: summary of results

This section presents the steps to identify basic, mission-oriented and applied research. Following the literature, we first present the patent indicators adopted in the analysis (Sect. 4.1), to then move to discuss their interpretation in the context of quest for fundamental understanding and consideration of use according to Stokes (Sect. 4.2). Finally, we present the methodology for attributing patent indicators to basic, mission-oriented and applied research (Sect. 4.3) and comment on the results (Sect. 4.4). Figure 4 summarise the results of the classification of indicators into the three Stokes’ quadrants.

## Research focus: patent indicators adopted in the analysis

To study how the focus or content of (patented) university research output has evolved through time, we adopt a variety of indicators from the literature. We build on information available from patent documents and offer detailed descriptions of the knowledge components of inventions as expressed by the different International Patent Classification (IPC) classes listed in a patent, the knowledge sources in the reference list, and the knowledge spillovers, described as the list of patents citing the focal patent. These indicators are constructed using information retrieved from Patstat Global or gathered from the OECD,^9^ and more specifically from the OECD Patent Quality Indicators Database (Squicciarini et al., 2013) and the OECD Citations Database (Webb et al., 2005). The various indicators employed in the analysis have been selected from the literature on university patents as described in Sect. 2.3: originality, generality, non-patent literature, forward citations, citation lag, opposition, patent scope, novelty, and family size. The next sections briefly describe the indicators and their relevance to the aims of this work.

*Originality* The originality index has often been employed as a measure of basic research and is therefore commonly adopted to study university patents. Used for the first time by Trajtenberg et al. (1997), the originality indicator identifies the dispersion of prior knowledge across different technologies. It is built on the IPCs of backward citations, that is, a Herfindahl–Hirschman concentration index of 4-digit IPCs upon which the focal patent draws on. It ranges from 0 to 1, where values closer to 1 indicate that the invention builds on a large variety of different technological fields and is therefore more original. We draw values of this indicator from the OECD Patent Quality Indicator dataset, which has pre-built values for all EPO patents, calculated as in Squicciarini et al. (2013).

*Generality* The generality index is very similar to the originality index but focuses on subsequent inventions rather than on knowledge sources. Trajtenberg et al. (1997) use this measure to represent university patents’ extensions of follow-up technical advantages across technological fields. Based on forward citations, it is a Herfindahl–Hirschman concentration index of 4-digit IPCs citing patents draw upon. It ranges from 0 to 1, where values closer to 1 indicate that the focal patent is a source of knowledge for a great variety of different technological fields. It is a measure of pervasiveness of the invention (Barbieri et al., 2020). We draw values of this indicator from the OECD Patent Quality Indicator dataset, which has pre-built values for all EPO patents, calculated as in Squicciarini et al. (2013).

*Non-patent literature* Inventions citing non-patent literature are considered to be closer in content to academic research, compared to inventions that do not cite any non-patent reference. Various works adopt this indicator to study the proximity between the knowledge content of the patent and academic research (e.g. Callaert et al., 2006, 2014; Sapsalis and van Pottelsberghe de la Potterie, 2007). The source of this information is the OECD Patent Quality Indicators dataset. We build a dummy indicator of non-patent literature that takes a value of 1 if the patent cites at least one non-patent document and 0 otherwise (Rizzo et al., 2020).

*Forward citations (first 3 years & total)* Forward citations is the most common indicator adopted to proxy patent quality. The underlying idea is that citing a patent represents building on the specific knowledge of that patent; accordingly, forward citations show the degree of contribution to further invention activities. Forward citations have been used to proxy several characteristics, such as value (Hall et al., 2005; Sapsalis et al., 2006), diffusion or knowledge spillovers generated (Sorenson and Felming, 2004; Dechezleprêtre et al., 2017), radicalness (Dahlin & Behrens, 2005). Forward citations is also the most commonly used indicator to compare university and firm patents (e.g. Sampat et al., 2003; Sterzi et al., 2019; Thursby et al., 2009; Trajtenberg et al., 1997). As detailed in Sect. 2.3, studies show different patterns of patent forward citations, so we build two variables of forward citation. The first one measures forward citations in the first 3 years after filing and captures the short-term impact of the invention. The second variable counts the overall number of forward citations—while this may suffer from truncation in the last years of our timeframe, it can still capture the longer-term impact of the invention as a seed for subsequent inventions up to the late 2000s. Both of these indicators are directly gathered from the OECD Citations Database.

*Citation lag* The temporal dynamic of citations has been acknowledged as relevant by several studies. Sampat et al. (2003) show that university patents receive more citations than industry patents, but only after a number of years. In line with this, Czarnitzki et al. (2011) adopt a measure of basicness referring to the time required for an invention to gain its first citation. We source this information from the OECD Citations Database and calculate the number of months before the first citation for every patent in our sample.

*Oppositions* This indicator has been adopted to proxy the basicness of research (Czarnitzki et al., 2009). By looking at opposition procedures initiated at the European Patent Office, we can then assess the degree of inventive gap between patented inventions, as perceived by the owners of potentially rival technologies. Information on opposition has been also employed to measure the value of a patent (Harhoff et al., 2003). We build a dummy indicator of opposition that takes a value of 1 if the patent received at least one opposition, and 0 otherwise. The opposition indicator is built from information in the OECD Citations Database.

*Patent scope.* Patent scope is the count of separate 4-digit IPC classes of a patent: the higher the number of distinct technological components (i.e. IPCs), the higher will be the breadth of the invention (Lerner, 1994). High levels of patent scope are associated with a higher value of inventions (Shane, 2001) and greater complexity (Barbieri et al., 2020). A higher degree of complexity also characterises patents stemming from research outputs that are more abstract and synthesise more information (Arora & Gambardella, 1994) compared to private research outputs. We draw values of this indicator from the OECD Patent Quality Indicator dataset, which has pre-built values for all EPO patents, calculated as in Squicciarini et al. (2013).

*Novelty* We employ the indicator of novelty in recombination proposed by Verhoeven et al. (2016), which identifies the novelty of a patent when two 8-digit IPC classes are combined for the first time. The index is built by comparing all possible pairs of IPC classes in a patent with all possible pairs in previous patents. The indicator is built as a dummy variable taking a value of 1 if the patent displays at least two IPCs never combined before. Following this method, university patents in the technological chemistry sector have been found to be more novel than private patents (Rizzo et al., 2020).

*Family Size* Together with citations and opposition, family size is a measure of patent value (Harhoff et al., 2003). Family size counts the distinct patent offices where an application has been made: the higher the number of jurisdictions in which a patent is protected, the higher its presumed value. The concept of patent family can also be extended to group together all documents pertaining to the same technology (EPO, 2017). We rely on the indicator provided by the OECD Patent Quality Indicator dataset, which is based on the INPADOC identifier of the EPO (EPO, 2017).

### Identification of basic, mission-oriented and applied research in patents

Starting from Stokes’ framework, the focus of research activities can be defined according to two dimensions: consideration of use and quest for fundamental understanding. As introduced in Sect. 2.2, Stokes identifies a two-by-two matrix where research activities with a focus on the quest for fundamental understanding alone are defined as pure basic research (*à la* Bohr), research activities that have both a consideration of use and a quest for fundamental understanding are defined as mission-oriented research (*à la* Pasteur), and research activities that have a consideration of use only are defined as pure applied research (*à la* Edison). To describe the evolution of the focus of publicly funded research (Sect. 5), we first need to assign patent indicators to Stokes’ quadrants and to do so, we compare university, university-transferred, and corporate patents.

We start from the assumption that a company will acquire university patents if their knowledge content is readily exploitable. Therefore, if a university-owned patent has short-term applicability and explicit problem-solving characteristics, its knowledge content explicitly manifests a consideration of use. As a consequence, university-transferred patents can be considered those with positive consideration of use, compared to university patents not transferred to industry. Likewise, to identify research more directly associated with the quest for fundamental understanding, we compare university-transferred patents with industry patents. It is reasonable to assume that industry will acquire university-patented research outputs that are needed but that cannot be autonomously produced. This suggests that the characteristics distinguishing university-transferred patents from industry patents represent the quest for fundamental understanding, e.g. codified research that is exploitable in the market but which the market alone does not or cannot produce.

The empirical exercise enables to assign to the pure basic research quadrant those patent indicators representing research features of university patents that industry does not finds useful. The mission-oriented basic research quadrant includes patent indicators representing research features that industry finds useful and does not produce on its own: these features can be found only in university patents that are traded to industry and not in university patents that remain property of university. The pure applied research quadrant includes patent indicators representing research content characteristics that are found only in the university patents that are acquired by industry (and not in university non-traded patents) and that industry also produces on its own.

### Methodology

The purpose of this section is to empirically compare university-transferred patents with university non-transferred patents in order to identify indicators that characterise research content with a consideration of use and to compare university-transferred patents with industry patents to identify indicators that characterise research content that demonstrates a quest for fundamental understanding.

To do so, we specify the following model:

$$Y_{i} = 1\left( {\alpha_{0} + \theta A_{i} + \mathop \sum \limits_{j = 1}^{J} \beta_{j} X_{i}^{j} + \mathop \sum \limits_{c = 1}^{C} \delta_{c} tech\;field_{i}^{c} + \mathop \sum \limits_{k = 1}^{K} \lambda_{k} geo_{i}^{k} + \mathop \sum \limits_{t = 1}^{T} \gamma_{t} time_{i}^{t} + \varepsilon_{i} > 0} \right),$$

where $Y_{i}$ is a binary variable identifying factors explaining the consideration of use or the factors associated with a fundamental quest. In the first model, we only consider university-owned patents and identify the ownership change such that it assumes the value of 0 if the patent is non-traded or the value of 1 if the patent is transferred at least once. In so doing, we estimate the factors associated with the consideration of use of the research. In the second model, we compare university-owned transferred patents with industry patents, such that $Y_{i}$ takes the value of 1 if the university patent is transferred and 0 if the patent is an industry patent—estimating the factors associated with research with a fundamental quest.

In each model we test, separately, the impact of a given patent indicator, $A$. Our key regressors are originality, generality, non-patent literature, novelty, patent scope, opposition, family size, 3-years and total forward citations, and citation lag. $X_{i}^{j}$ is a set of control variables accounting for the patent feature $j$ of patent $i$. Specifically, we control for backward citations (*Bwd Cits*), collected from the OECD Patent Quality Indicator dataset (Squicciarini et al., 2013), the number of 8-digit IPC classes (*N Ipc*), the number of inventors (*N Inventors*), and a dummy variable identifying the presence of multiple applicants (*N Applicant*), using information collected from the OECD Regpat dataset. We also include the number of universities that applied for at least one patent in each time period (*N Uni app*), derived from Patstat Global. This variable controls for those universities that might apply for a patent for the first time motivated more by third mission and evaluation incentive mechanisms rather than the commercial value of their research (Della Malva et al., 2013).

We also control for technological-field fixed effects ($tech field_{i}^{c}$), country dummies ($geo_{i}^{k}$), and time effects ($time_{i}^{t}$). In particular, $time_{i}^{t}$ is a dummy variable that takes the value of 1 if patent application $i$ is included in the time window $t$ (t = 1978–1985, 1986–1990, 1991–1995, 1996–2000, 2001–2005, 2006–2010, 2011–2015). $\varepsilon_{i}$ is an independently and identically distributed error term.

### Results

Table 1 reports descriptive statistics of the variables, and Table 2 presents the correlation matrix. Results of the logit regressions are presented in Tables 3, 4, and 5.^10^ The indicators of originality and generality do not significantly associate to the quest for fundamental understanding, nor to the consideration of use, therefore we exclude them for the rest of the analysis.^11^

**Table 1:** Descriptive statistics

Variable Name	Obs	Mean	SD	Min	Max
Originality	104,3011	0.653	0.246	0	1
Generality	365,585	0.326	0.281	0	1
Non-pat lit	1,111,834	0.230	0.421	0	1
Novelty	1,111,834	0.043	0.203	0	1
Patent scope	1,111,834	1.795	1.061	1	33
Opposition	489,509	0.061	0.239	0	1
Family size	1,111,834	5.239	3.930	1	57
Fwd cits tot	1,111,834	2.830	4.666	0	297
Fwd cits 3y	1,111,834	0.805	1.818	0	108
Citation lag	731,660	53.972	52.337	0	482
Bwd cits	1,111,834	4.940	6.457	0	396
N Ipc	1,111,834	3.536	3.494	1	99
N inventors	1,111,834	2.385	1.662	0	99
N applicant	1,111,834	0.061	0.239	0	1
N Univ app	1,111,834	1,026.978	594.415	183	1774

**Table 2:** Correlation matrix

Variables	(1)	(2)	(3)	(4)	(5)	(6)	(7)	(8)	(9)	(10)	(11)	(12)	(13)	(14)
(1) Originality	1.00													
(2) Generality	0.29	1.00												
(3) Non-pat lit	0.08	0.11	1.00											
(4) Novelty	0.09	0.11	0.00	1.00										
(5) Patent scope	0.29	0.37	0.11	0.37	1.00									
(6) Opposition	0.04	0.05	0.00	0.01	0.03	1.00								
(7) Family size	0.09	0.14	0.11	0.07	0.22	0.08	1.00							
(8) Fwd cits tot	0.08	0.19	0.08	0.07	0.18	0.13	0.30	1.00						
(9) Fwd Cits 3y	0.10	0.15	0.10	0.02	0.13	0.06	0.24	0.69	1.00					
(10) Citation lag	−0.10	−0.09	−0.08	0.01	−0.06	−0.03	−0.09	−0.25	−0.37	1.00				
(11) Bwd cits	0.17	0.03	0.07	0.00	0.03	0.06	0.05	0.06	0.09	−0.06	1.00			
(12) N Ipc	0.21	0.23	0.13	0.25	0.62	0.03	0.33	0.26	0.20	−0.09	0.03	1.00		
(13) N inventors	0.13	0.11	0.15	0.01	0.13	0.04	0.18	0.13	0.17	−0.12	0.10	0.18	1.00	
(14) N applicant	0.03	0.03	0.07	0.00	0.03	0.00	0.04	0.04	0.04	−0.03	0.01	0.04	0.11	1.00
(15) N Univ app	0.14	−0.08	0.05	−0.09	−0.11	−0.03	−0.14	−0.16	0.02	−0.27	0.16	−0.15	0.12	0.00

**Table 3:** Pure basic research (Bohr)

	(1)	(2)	(3)	(4)
	Cons. of use	Fund. quest	Cons. of use	Fund. quest
Non-pat lit	−0.0514	1.423***		
	(0.0519)	(0.0485)		
Novelty			0.0653	0.503***
			(0.113)	(0.100)
Bwd cits	0.00662	−0.0698***	0.00673	−0.0734***
	(0.00283)	(0.00787)	(0.00282)	(0.00918)
N Ipc	0.0308***	−0.000795	0.0296***	0.000375
	(0.00585)	(0.00466)	(0.00607)	(0.00453)
N inventors	0.0326**	0.0722***	0.0319**	0.0833***
	(0.0116)	(0.00734)	(0.0116)	(0.00698)
N applicant	0.703***	2.060***	0.701***	2.115***
	(0.0521)	(0.0404)	(0.0520)	(0.0404)
N Univ app	−9.65e-05	0.0016***	−0.00010	0.0016***
	(0.0001)	(0.0001)	(0.0001)	(0.0001)
Observations	22,920	1,091,828	22,920	1,091,828
chi2	1025	11,443	1025	10,933

Robust standard errors in parentheses; time, technological field and country dummies included but not displayed; *p < 0.05, **p < 0.01, ***p < 0.001

**Table 4:** Mission-oriented basic research (Pasteur)

	(1)	(2)	(5)	(6)	(7)	(8)
	Cons. of use	Fund. quest	Cons. of use	Fund. quest	Cons. of use	Fund. quest
Patent scope	−0.0482*	0.110***				
	(0.0245)	(0.0211)				
Family size			0.0582***	−0.0975***		
			(0.00640)	(0.00612)		
Fwd cits tot					0.0334***	0.0188***
					(0.00357)	(0.00235)
bwd cits	0.00672	−0.0729***	0.00589	−0.0697***	0.00492	−0.0769***
	(0.00283)	(0.00916)	(0.00292)	(0.00921)	(0.00294)	(0.00934)
N Ipc	0.0409***	−0.0128*	0.0204***	0.0156***	0.0218***	−0.00160
	(0.00786)	(0.00574)	(0.00602)	(0.00410)	(0.00604)	(0.00453)
N inventors	0.0325**	0.0811***	0.0291	0.101***	0.0250	0.0763***
	(0.0116)	(0.00702)	(0.0118)	(0.00780)	(0.0118)	(0.00710)
N applicant	0.705***	2.114***	0.682***	2.111***	0.705***	2.111***
	(0.0521)	(0.0404)	(0.0523)	(0.0405)	(0.0524)	(0.0404)
N Univ app	−0.000108	0.00162***	−4.42e-05	0.00152***	−3.62e-05	0.00163***
	(0.000129)	(0.000124)	(0.000131)	(0.000124)	(0.000131)	(0.000124)
Observations	22,920	1,091,828	22,920	1,091,828	22,920	1,091,828
chi2	1029	10,893	1097	11,164	1107	10,976

Robust standard errors in parentheses; time, technological field and country dummies included but not displayed; *p < 0.05, **p < 0.01, ***p < 0.001

**Table 5:** Pure applied research (Edison)

	(1)	(2)	(3)	(4)	(5)	(6)
	Cons. of use	Fund. quest	Cons. of use	Fund. quest	Cons. of use	Fund. quest
Fwd cits 3y	0.0657***	0.00595				
	(0.00965)	(0.00862)				
Citation lag			−0.00325***	−0.00123		
			(0.000839)	(0.000664)		
Oppositions					0.593**	−0.410
					(0.185)	(0.165)
Bwd cits	0.00543	−0.0733***	0.00455	−0.0765***	0.00660	−0.0721***
	(0.00290)	(0.00922)	(0.00332)	(0.0102)	(0.00283)	(0.00916)
N Ipc	0.0260***	0.00363	0.0286***	0.00466	0.0300***	0.00444
	(0.00595)	(0.00451)	(0.00635)	(0.00466)	(0.00584)	(0.00439)
N inventors	0.0272*	0.0819***	0.0330*	0.0706***	0.0318**	0.0831***
	(0.0118)	(0.00711)	(0.0136)	(0.00813)	(0.0116)	(0.00698)
N applicant	0.703***	2.113***	0.701***	1.991***	0.704***	2.113***
	(0.0522)	(0.0404)	(0.0617)	(0.0479)	(0.0521)	(0.0404)
N Uni app	−0.000121	0.00160***	−0.000161	0.00156***	−9.79e-05	0.00160***
	(0.000129)	(0.000124)	(0.000138)	(0.000130)	(0.000130)	(0.000124)
Observations	22,920	1,091,828	14,110	719,646	22,920	1,091,828
chi2	1057	10,944	684.6	7603	1031	10,932

Robust standard errors in parentheses; time, technological field and country dummies included but not displayed; *p < 0.05, **p < 0.01, ***p < 0.001

Table 3 shows the regression output for patent indicators associated to a quest for fundamental understanding but without consideration of use, showing the indicators that characterise pure basic research *à la* Bohr: novelty and non-patent literature. From Table 3 we can see that novelty and non-patent literature significantly associate only to the quest for fundamental understanding, showing a positive sign. University patents that are transferred to industry, compared to industry patents, show significantly higher probability of being novel and of citing non-patent literature. Conversely university non-transferred and university-transferred patents do not show any statistical difference in terms of novelty and non-patent literature. These results lead us to consider the indicators of novelty and of non-patent literature to represent pure basic research features.

Table 4 presents results for patent indicators that determine both consideration of use and a fundamental quest, thus depicting mission-oriented basic research *à la* Pasteur. The indicators populating this quadrant are patent scope, family size, and total forward citations. Table 4 illustrates that these features show differences between university transferred patents and both university non-transferred patents and industry patents. More specifically, we note that university transferred patents display less patent scope than university non-transferred patents and higher patent scope than industry patents. Family size of university transferred patents is averagely higher than university non-transferred patents but lower than industry patents. Conversely, university transferred patents show averagely higher total citations than both university non-transferred patents and industry patents.

Finally, Table 5 reports the patent indicators that distinguish consideration of use but not a quest for fundamental understanding—3-years forward citations, citation lag and opposition—thus depicting applied research *à la* Edison. We can see from Table 5 that university transferred patents exhibit higher number of short-term forward citations, higher probability of receiving an opposition and lower citation lag compared to university non-transferred patents. Conversely, there is no significant difference between university transferred and industry patents across these features.

This exercise, by comparing university transferred patents with both university non-transferred patents and with industry patents allows us to classify patent indicators based on their association with the consideration of use and quest for fundamental understanding categories, respectively, of the invention content. According to these results, following Stokes’ framework, we observe that novelty and non-patent literature are significant proxies of pure basic research; patent scope, family size and total citations of mission oriented basic research; and 3 years forward citations, citation lag and opposition of pure applied research. The following section will adopt these indicators to represent pure basic, mission-oriented and pure applied research and monitor their evolution to assess changes in the content, or focus, of university patents along time.

## The evolution of university and industry research knowledge content from 1978 to 2015

### Methodology

The previous section presented the patent indicators adopted to describe different research focus of university patents. Building on this, the current section focuses on the evolution of publicly funded university research output vis-a-vis industry research output. Accordingly, this section looks at how the pure basic, mission-oriented, and pure applied focus of (patented) university research evolved from 1978 to 2015.

To do so, we adopt the following model:

$$Y_{i}^{A} = \mathop \sum \limits_{t = 1}^{T} \left[ {\alpha_{t} time_{i}^{t} + \beta_{t} \left( {time_{i}^{t} *UNIV_{i} } \right)} \right] + \mathop \sum \limits_{j = 1}^{J} \gamma_{j} X_{i}^{j} + \mathop \sum \limits_{c = 1}^{C} \delta_{c} tech field_{i}^{c} + \mathop \sum \limits_{k = 1}^{K} \lambda_{k} geo_{i}^{k} + \varepsilon_{i} ,$$

where $Y_{i}^{A}$ represents indicator $A$ of patent $i$. Indicators $A$ employed as dependent variables are those characterizing the content of basic (non-patent literature, novelty), mission-oriented (patent scope, family size, total forward citations), and applied research (3-years forward citations, citation lag, oppositions) (these are discussed in Sect. 4.2). $UNIV_{i}$ is a dummy variable taking the value of 1 if patent $i$ is university-owned and 0 otherwise. $time_{t}$ is a dummy variable assuming the value of 1 if patent $i$ was applied for within the stacked time window $t$ ($t$ = 1978–1985, 1986–1990, 1991–1995, 1996–2000, 2001–2005, 2006–2010, 2011–2015).^12^$X_{i}^{j}$ is a control variable accounting for patent $j$ features, namely backward citations, number of 8-digit IPCs, number of inventors, presence of multiple applicants and the number of universities that applied for at least one patent. Moreover, we include a set of dummy variables capturing the specific features of each technological field $c$ of patent $i$ ($tech field$) and to control for heterogeneous effects across geographical areas $k$ ($geo$). Finally, $\varepsilon_{i}$ is an independently and identically distributed error term.

**Fig. 5:**
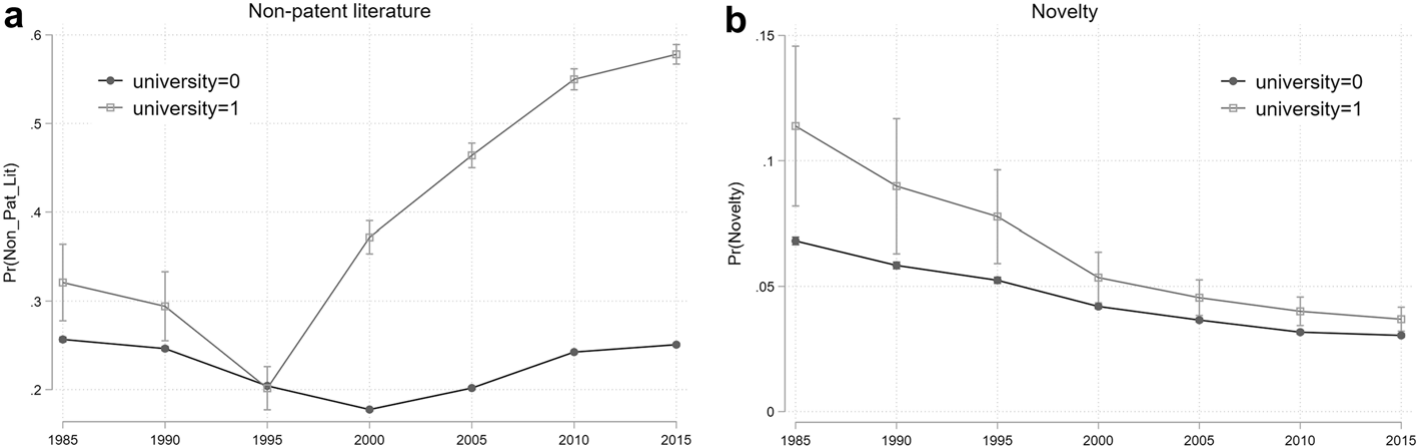
Predicted probability of university versus industry patents: basic research indicators

**Fig. 6:**
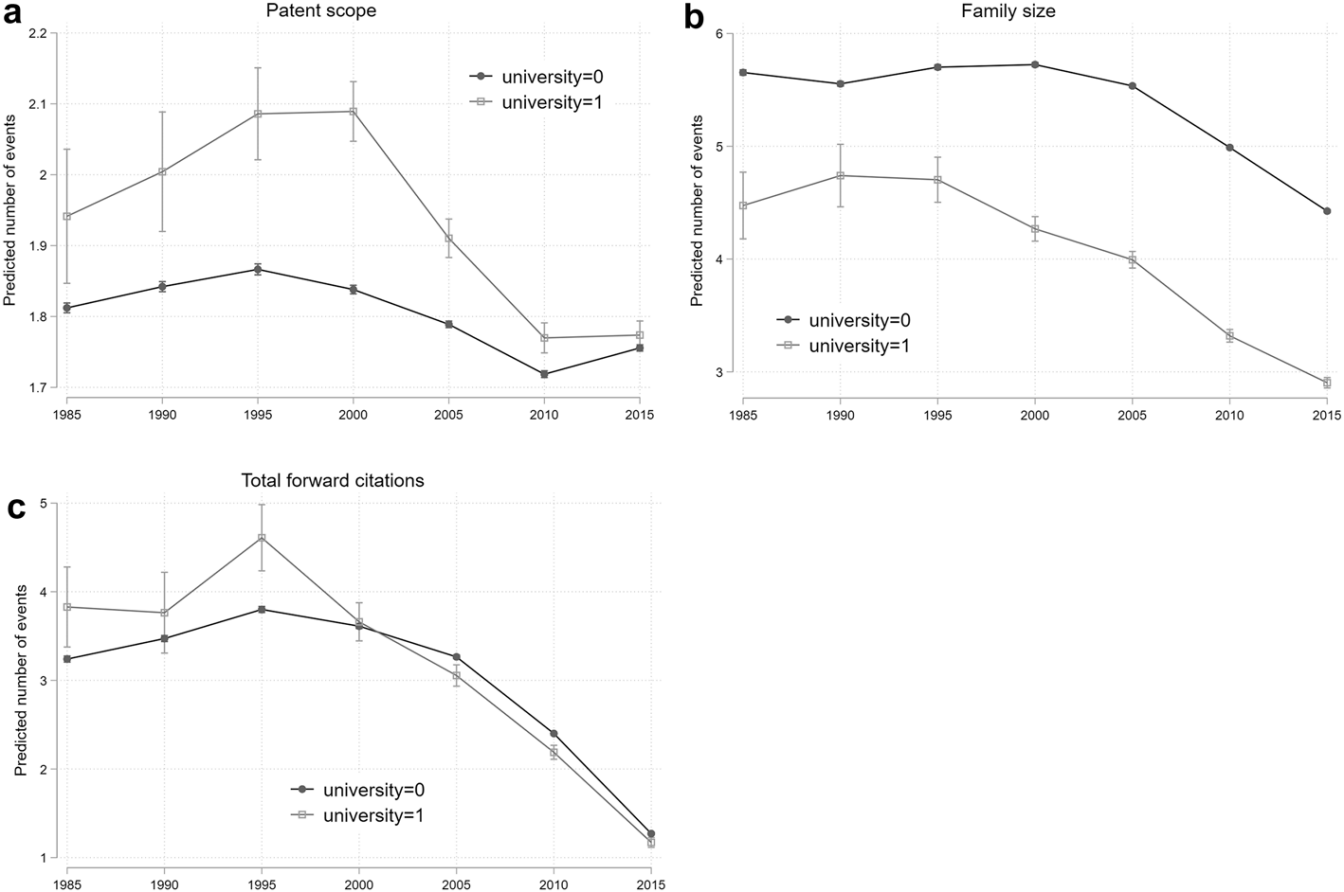
Predicted probability of university versus industry patents: Mission-oriented research

**Fig. 7:**
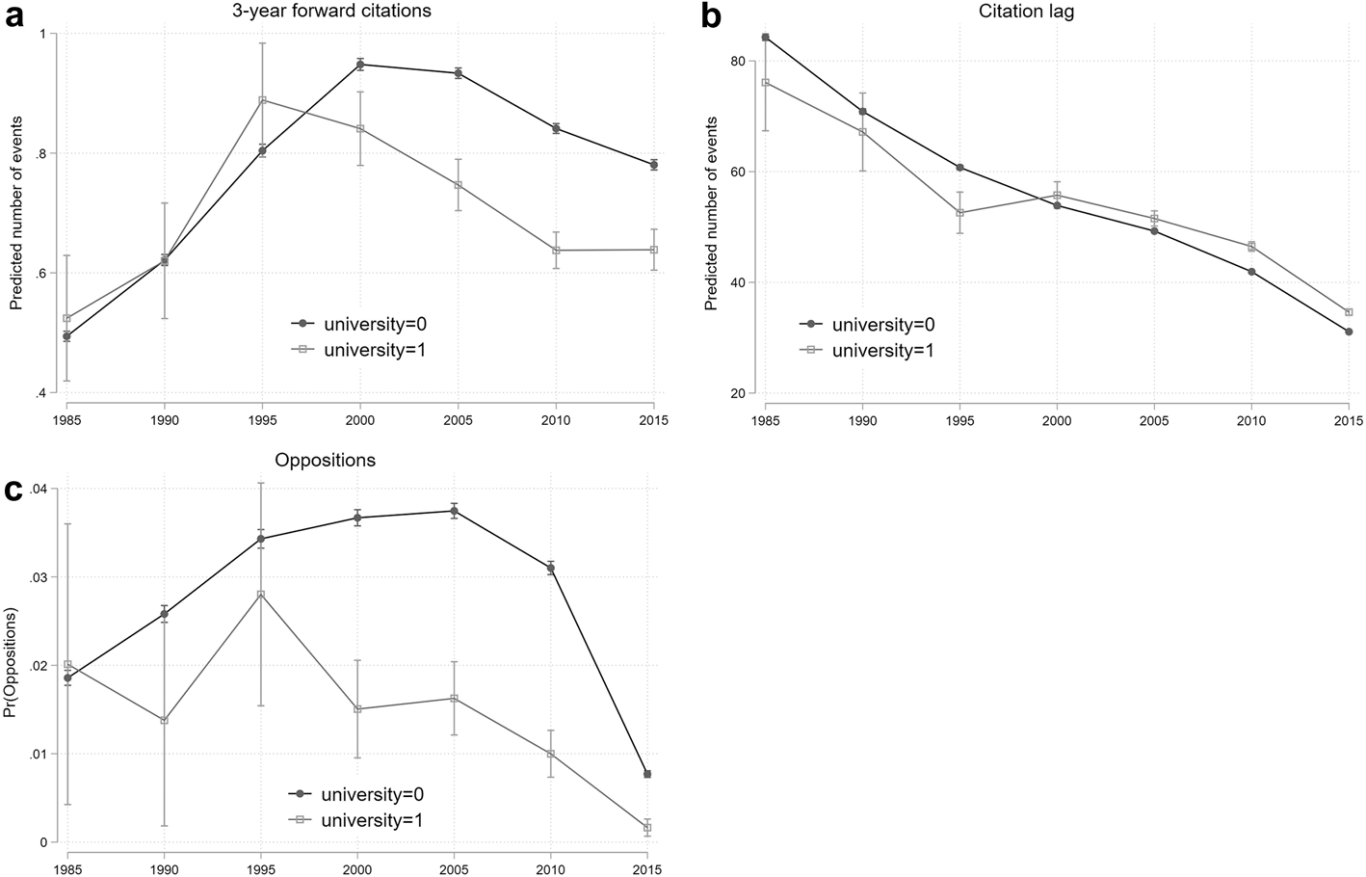
Predicted probability of university versus industry patents: Applied research

The choice of estimation method depends on the nature of the indicators. We employ a logit model when the dependent variable is binary, so for the novelty in recombination index, the non-patent literature indicator, and oppositions. Conversely, forward citations, oppositions, family size, patent scope, and citation lag are countable variables and thus we implement Poisson regressions. All specifications include robust standard errors. It is worth highlighting that in the presence of interaction effects and categorical variables, the interpretation of estimation results can be challenging.^13^ In order to properly assess changes in patent features over the time period considered and test whether there are differences between university- and company-owned patents, we compute marginal effects measuring changes in predicted probabilities across groups at different time intervals. Finally, we show a graphical representation of the predicted probability patterns over time (Figs. 5, 6, 7). Greene (2010) highlights how a graphical representation of interaction effects may be an effective solution to inform model implications.

### Results

We present three tables, one for each of the Stokes quadrants. Tables 6, 7, 8 report the estimated coefficients showing how university research evolved in comparison to industry research over the 1978–2015 period. We then calculate the marginal effects and provide a graphical representation quantifying different probabilities of observing pure basic research features between university-owned and company patents at different time intervals.^14^ Panels (a) and (b) in Figs. 5, 6, 7 show the predicted probability of observing an improvement in patent characteristics for university and industry patents during the period analysed, with a 95% confidence interval level.

**Table 6:** University versus industry patents: Basic research

	(1)	(2)
	Non-Pat Lit	Novelty
1978–1985	0.414**	0.624***
	(0.135)	(0.180)
1986–1990	0.318*	0.514**
	(0.127)	(0.186)
1991–1995	−0.0208	0.464**
	(0.0987)	(0.147)
1996–2000	1.316***	0.281*
	(0.0553)	(0.113)
2001–2005	1.629***	0.252**
	(0.0388)	(0.0930)
2006–2010	1.789***	0.267**
	(0.0330)	(0.0847)
2011–2015	1.883***	0.221**
	(0.0310)	(0.0772)
Bwd cits	0.0389***	0.00248**
	(0.000638)	(0.000817)
N Ipc	0.0117***	0.225***
	(0.000737)	(0.00207)
N inventors	0.0692***	−0.0327***
	(0.00158)	(0.00371)
N applicant	0.166***	0.0414
	(0.0103)	(0.0216)
N Uni app	−2.56e-05***	−0.000587***
	(6.78e-06)	(1.23e-05)
Observations	1,111,834	1,111,834
chi2	183,291	28,182

Robust standard errors in parentheses; time, technological field and country dummies included but not displayed; *p < 0.05, **p < 0.01, ***p < 0.001

**Table 7:** University versus industry patents: Mission-oriented research

	(1)	(2)	(3)
	Patent scope	Family size	Fwd cits tot
1978–1985	0.0689**	−0.234***	0.167**
	(0.0249)	(0.0338)	(0.0603)
1986–1990	0.0843***	−0.158***	0.0807
	(0.0215)	(0.0299)	(0.0618)
1991–1995	0.111***	−0.192***	0.193***
	(0.0160)	(0.0218)	(0.0416)
1996–2000	0.128***	−0.294***	0.0134
	(0.0104)	(0.0131)	(0.0302)
2001–2005	0.0656***	−0.326***	−0.0666**
	(0.00739)	(0.00951)	(0.0204)
2006–2010	0.0293***	−0.407***	−0.0923***
	(0.00601)	(0.00880)	(0.0187)
2011–2015	0.0103	−0.421***	−0.0814***
	(0.00567)	(0.00825)	(0.0245)
Bwd cits	0.00137***	0.00225***	0.00848***
	(7.43e-05)	(9.92e-05)	(0.000229)
N Ipc	0.0465***	0.0207***	0.0382***
	(0.000632)	(0.000243)	(0.000618)
N inventors	0.00491***	0.0407***	0.0718***
	(0.000528)	(0.000873)	(0.00496)
N applicant	0.0153***	0.0178***	0.111***
	(0.00287)	(0.00271)	(0.00731)
N Uni app	−1.99e-05***	−0.000154***	−0.000588***
	(1.47e-06)	(1.68e-06)	(5.14e-06)
Observations	1,111,834	1,111,834	1,111,834
chi2	150,806	217,850	104,603

Robust standard errors in parentheses; time, technological field and country dummies included but not displayed; *p < 0.05, **p < 0.01, ***p < 0.001

**Table 8:** University versus industry patents: Applied research

	(1)	(2)	(3)
	3-y Fwd cits	Citation lag	Oppositions
1978–1985	0.0594	−0.102	0.0822
	(0.102)	(0.0586)	(0.415)
1986–1990	−0.00209	−0.0536	−0.646
	(0.0795)	(0.0536)	(0.452)
1991–1995	0.100	−0.144***	−0.211
	(0.0549)	(0.0363)	(0.239)
1996–2000	−0.120**	0.0340	−0.923***
	(0.0377)	(0.0222)	(0.192)
2001–2005	−0.223***	0.0451**	−0.866***
	(0.0297)	(0.0138)	(0.134)
2006–2010	−0.277***	0.103***	−1.164***
	(0.0248)	(0.00959)	(0.138)
2011–2015	−0.201***	0.108***	−1.556***
	(0.0277)	(0.00809)	(0.303)
Bwd Cits	0.00686***	−0.00514***	0.0131***
	(0.000222)	(0.000218)	(0.000511)
N Ipc	0.0386***	−0.0271***	0.0361***
	(0.000695)	(0.000389)	(0.00119)
N Inventors	0.0799***	−0.0309***	0.0563***
	(0.00610)	(0.000698)	(0.00327)
N Applicant	0.140***	−0.0449***	0.0509
	(0.00994)	(0.00451)	(0.0251)
N Uni app	0.000287***	−0.000627***	−0.000565***
	(7.11e-06)	(2.51e-06)	(2.20e-05)
Observations	1,111,834	731,660	1,111,834
chi2	60,927	146,605	13,444

Robust standard errors in parentheses; time, technological field and country dummies included but not displayed; *p < 0.05, **p < 0.01, ***p < 0.001

#### Basic research

Non-patent literature is a distinctive characteristic of university research compared to company inventions, with the exception of the 1991–1995 time period. Moreover, the university coefficient displays a considerable increase in magnitude: in the early eighties, a university patent had a 32 percent probability of referencing non-patent literature, against 25 percent for an industry patent, while between 2010 and 2015 the probability of a university patent citing non-patent references was 58 percent, which is more than 30 percent higher than the probability for a corporate patent. The difference in the probability of a university patent citing non-patent references was significantly higher in 2015 than in 1985 (chi2 = 144.1).

With regards to novelty in recombination index, university patents appear to be more novel than industry patents in all time periods (the coefficient seems to reduce in size, but the reduction is not significant). Panel (b) in Fig. 5 illustrates this pattern.

Overall, the analysis suggests that university research has a distinctive basic research focus: non-patent literature increases its weight in university-owned patents compared to industry patents along the timeframe analysed, while novelty shows consistently higher levels for university patents.

#### Mission oriented research

For the indicators characterising mission-oriented basic research, we report the main results in Table 7 (estimated coefficients), while Fig. 6 (panels a to c) graphically illustrates the patterns of predicted probabilities comparing university and company patents over the analysed period. The patent-scope indicator, a measure of the breadth or complexity of inventions, shows that university patents display a greater scope than industry patents over the whole timeframe analysed, except for the last time period. More specifically, university patent scope increases compared to corporate patent scope from the early eighties to the late nineties, but from 2000s onwards the gap starts to reduce, reaching a statistically non-different level for the 2011–2015 period.^15^ In the period with the highest value (1996–2000), university patents have a predicted scope of 2.1 against a scope of 1.8 for industry patents. In the late 2000s, the difference reduces to 0.05, while there is no difference in the 2011–2015 period. Figure 6, panel (a), clearly illustrates the pattern of convergence of the average patent scope of inventions from universities and companies since the 2000s.

Family size (Table 7, column 2, and Fig. 6, panel b) presents a much lower magnitude for university patents than for industry patents. This was expected, as we saw from Table 4 that transferred university patents have larger family size than the university patent average, but lower than industry patents. Such a difference does not statistically change throughout the timeframe considered. With reference to the 2011–2015 period of analysis, the average university patent family size is 2.9, against an average company patent family size of 4.4.

The final indicator proxying the mission-oriented content of research is the number of total forward citations (Table 7, column 3, and Fig. 6, panel c). In this case, we can see that up to the year 2000 university patents received more or a similar number of citations compared to industry patents, while from 2005 onwards university patents start being cited less than company patents: even though the difference in size remains small, it is significant at the 1% level (in 2000–2005, the predicted number of citations for industry patents is 3.26 while that of university patents is 3.05). Even acknowledging the presence of truncation bias (that allows us to consider this indicator safely only up to mid-2000s), we can argue that the average frequency of total forward citations in university patents decreased from year 2000.

We therefore see that the mission-oriented content of university research shows complex patterns, with two out of three indicators indicating this activity has decreased in universities in more recent years. Specifically, the difference between university and industry patents in terms of patent scope increased up to the early 2000s and then decreased, indicating that this feature of mission-oriented content is found mostly in university patents only up to the early 2000s, while afterwards the level of university patents’ scope is the same as industry patents. Similarly, the average number of total forward citations show a reduction in university patents around 2000, reaching similar level of industry patents, indicating that this mission-oriented feature has reduced in university research. Overall, we document a decrease from the mid-2000s onwards for most of the features of mission-oriented basic research.

#### Applied research

Finally, we compare how the content of pure applied research has evolved in university compared to industry. Table 8 shows the estimated coefficients when we focus on forward citations and citation lag. Analysing the trends of the predicted probabilities (Fig. 7, panels a to c), we observe an average decrease in universities’ pure applied research patents with respect to industry’s average value. Specifically, the three indicators show similar coefficients for both company and university patents at the beginning of the period, while from the late nineties (early 2000s for citation lag) onwards the pure applied research content in patented output becomes averagely lower in universities compared to companies. Note that lower citation lag (as well as lower oppositions) means higher content of applied research, therefore an increase in the average citation lag (oppositions) of university patents indicates a decrease in the applied research focus of patents. These three indicators may suffer from truncation bias, however the reduction trend seems to start early enough to allow the identification of a clear pattern: pure applied research content in university patents diminishes with respect to industry patents.

## Discussion

The current analysis aims to explore the evolution of the research focus, as measured by publicly funded patents, produced by universities in the last forty years. By analysing variations in indicators describing the knowledge content of university patents, we observe a shift from basic research with consideration of use (mission-oriented) towards basic research without consideration of use (basic research), as well as a decrease in applied research. Our results suggest that compared to industry, universities veered their efforts towards more basic research (a-la Bohr), and less mission-oriented (a-la Pasteur) and applied (a-la Edison) research. These results complement and extend the literature investigating these patterns in the private sector which revealed that industry is struggling to appropriate basic research from universities (Arora, 2018; 2020). Also, by distinguishing basic research in pure basic (without consideration of use) and mission-oriented (with consideration of use), we add to the literature concerned with the underestimation of returns to social value (Azoulay & Li, 2020; Calvert, 2006) unravelling the tensions between basic and applied research. Our findings support the view that adopting a basic versus applied research dichotomy is misleading and underrates the contribution of universities to knowledge production and value for society (Cockburn & Henderson, 1997; Marburger III, 2005). While previous works have highlighted such inefficiency with concerns to public R&D spending (Li et al., 2017; Marburger III, 2005), by adopting Stokes (1997) as a framework we unpack the focus of university patented research that has been publicly funded, to articulate the tensions in the division of labour between academia and industry (Nelson & Romer, 1996).

According to Stokes’, mission-oriented research (a-la Pasteur) represents science motivated by both a fundamental quest for understanding (proper of basic research) and consideration of use aimed at solving practical problems (proper of applied research). Our results suggest two dynamics. First, we find that for most of the period under consideration, patents a-la Pasteur are predominantly a feature of universities rather than industry. This is in line with scientists’ own views of the destination of use of their research, which they find difficult to disentangle between basic and applied, as it is often motivated by a combination of both aspects (Bentley et al., 2015; Gulbrandsen & Kyvik, 2010). Also, this result supports that the input of universities to societal needs should include measures of its translational capacity to industry (Calvert, 2006): research a-la Pasteur, as basic research motivated by a consideration of use, is crucial in this respect. A second result of our analysis is that, while still being characteristic of universities, mission-oriented research has been steadily declining since the late 1990s, in comparison to industry. This result sheds light on findings from the private R&D literature (Arora et al., 2018; Zahra et al., 2018): industry’s strategy of sourcing basic science directly from universities has increased over time, but its capacity to appropriate basic science has proved more challenging (Arora et al., 2020). In particular, ‘corporations turned to sourcing ideas and inventions from the outside, hoping to combine them with their downstream development and commercialization abilities. These hopes have not been fully realized, at least not yet’ (Arora et al., 2020, p. 86). The decline of universities’ mission-oriented research, the one more readily digestible in terms of industrial applications, might contribute to understand why the private sector is struggling to grapple with insourcing knowledge from universities.

Further to the decline in mission-oriented research, the capacity of industry to absorb basic research produced at university might have been also pushed by a shift in the intensity, as expressed by patent indicators, of basic research. Since 1975, universities have not just steadily increased their basic research focus, but defining features of patents a-la Bohr, such as novelty in recombination and non-patent literature, have become more specific of university basic research, compared to industry. In particular, non-patent literature, an indicator typically representing the proximity between scientific outputs and inventive activity (Callaert et al., 2014; Rizzo et al., 2020), has become increasingly distanced from industry’s (Fig. 5, panel a). In combination with the decline of more exploitable mission-oriented research, this might have impacted on the capacity of companies to absorb and apply basic research produced at universities for their developmental activities (Callaert et al., 2014; Meyer, 2000).

Finally, we note that while the private sector is focussing more on applied R&D spending (Arora et al., 2018), university-owned patents follow the opposite pattern, with applied research patents (a-la Edison) produced by universities steadily declining since 1978. These results seem to conflict with the policy changes of the last thirty years, focussed on increasing the impact of university research. In this respect, our work complements studies finding that changes to incentive mechanisms did not reduce the scientific production and impact of academic research (Godin & Gingras, 2000; Hicks & Hamilton, 1999). Indeed, changes to the funding rationale and the institutionalisation of third mission activities do not seem to have produced an increase in publicly funded applied research at universities (Perkmann et al., 2013), at least in comparison to industry.

## Conclusions

Our study makes two main contributions to the literature. First, we propose a method to classify basic, mission-oriented and applied research, by adopting a range of indicators used in the patent literature to assess patents’ basicness. By comparing industry patents, university patents and patents transferred from university to industry, we empirically disentangle basic research into basic research with and without consideration of use, allowing to overcome the basic versus applied dichotomy (e.g.: Czarnitzki et al., 2009; Trajtenberg et al., 1997). Second, we complement and extend existing literature by first, expanding the analysis to mission-oriented research and hence providing a more nuanced understanding of basic research motivated by consideration of use for society (Stokes, 1997), and second, offering for the first time a picture of how basic, applied and mission-oriented content of university publicly-funded and patented research has evolved across Europe in the last forty years. By taking university as our focus, we extend current literature that has looked at the evolution of basic and applied research from an industry angle (Arora et al., 2018; Zahra et al., 2018). Conversely, our work offers an overview of the division of labour between industry and university from the perspective of academia, arguably the main stakeholder in the production of basic research.

Our study is explorative in nature and, therefore, caution is needed in terms of implications. First, we proxy the content of public research exclusively with patents. While patents present many advantages in terms of consistency and robustness, they also suffer from significant issues. In particular, they neither represent the full range of university engagement activities (Perkmann et al., 2021) nor include the much broader range of knowledge-exchange channels of university activities (Hughes & Kitson, 2012; Marzocchi et al., 2023). Moreover, the increased trend in university patenting activity might be a consequence of third mission policies: while we seek to control for this in our empirical analysis, caution is needed in the interpretation of results. Future analyses could improve the robustness of these results by modelling policy changes more accurately (i.e.: through the introduction of specific incentive mechanisms/third mission policies) at the country level. It is also relevant to point out that patents exclude a vast share of knowledge produced in universities by disciplines whose content is subject to other forms of IPRs or no IPRs at all, such as the humanities and social sciences (Olmos-Penuela et al., 2014), and exacerbate issues of performance evaluation in science (Hallonsten, 2021). A final limitation is that while our data rely only on transferred patents, further analyses should discriminate between patents acquired and patents licenced by universities. Licences are a non-negligible part of universities’ strategies to commercialise their patents while retaining IP profits. Studies concerning U.S. universities have corroborate these patterns (Cavaggioli et al., 2020), but there is still a lack of evidence with respect to European countries.

Our results suggest that the division of labour between university and industry (Arora et al., 2019; Rosenberg & Nelson, 1994), measured through the comparison between university and industry patented research, has evolved. Accordingly, further analysis is needed to understand why this recalibration happened, how it might affect the appropriation of publicly funded resources (university research) and its impact on social value redistribution, e.g.: whether the current funding rationales and incentive systems are safeguarding industry appropriation mechanisms at the expense of society at large (Azoulay et al., 2009). By evidencing the decline of basic research content with consideration of use — e.g.: mission-oriented research—coming from universities, this work suggests there is a vacuum in the knowledge production of research *à la* Pasteur from the public sector. This is particularly relevant given that policymakers still regard basic research as vital for society and devote considerable resources to its funding (IMF, 2021). However, as our analysis points out, the division of labour between university and industry is changing and ‘we could end up with the kind of separation that we have avoided until now, with the Bohrs working in isolation from the Edisons, and with little work in the Pasteurs’ quadrant.’ (Nelson & Romer, 1996, pp. 10). Accordingly, policy makers should reflect on how the system of incentives introduced in the last thirty years, while supporting a broader engagement of academics with industry, has possibly reduced the returns to social value by universities. This in turn might affect the perception society holds with respect to academia and its contribution to progress and growth (Calvert, 2006; Cross et al., 2021).

## Footnotes

1 https://www.astrazeneca.com/media-centre/press-releases/2020/astrazeneca-and-oxford-university-announce-landmark-agreement-for-covid-19-vaccine.html. Accessed in May 2022.

2 https://www.ox.ac.uk/news/2020-11-23-oxford-university-breakthrough-global-covid-19-vaccine. Accessed in May 2022.

3 U.S. basic research expenditures by performing sector and source of funds (1953–2018). Data on 2017 and 2018 are not reported here as they are based on estimates. Accessed in September 2021 at https://ncses.nsf.gov/pubs/nsf21325#data-tables

4 Our sample of patents includes all publicly owned patents from universities, governments, and hospitals. University patents are the majority, thus, we use the terms public patent and university-owned patent interchangeably.

5 Our sample of university patents does not consider patents owned by companies in which an inventor is an academic scientist: these patents are an important share of academic staff patenting activity (e.g. Lissoni and Montobbio, 2015). However, the research underpinning these patents is privately funded, whereas we are interested in the research content of publicly funded research.

6 We perform robustness checks adopting different methods of selecting a patent application within each patent family, such as the average value according to the same procedure, or the value of the oldest patent application. They all return very similar results, and these are available from the authors upon request.

7 We identify this threshold in order to take into consideration all major European countries and compare sufficient number of patents, especially when dealing with transferred patents. Our results are robust to lower thresholds levels that include further countries.

8 Various works confirm this decrease, although some date it earlier than our results (e.g. Geuna and Rossi, 2011).

9 http://www.oecd.org/sti/inno/intellectual-property-statistics-and-analysis.htm.

10 We adopt a 5% P-value cut-off when investigating the consideration of use, while we adopt a more restrictive 1% P-value cut-off when investigating the fundamental quest because of the much larger sample size.

11 Results are available on request to authors.

12 As robustness checks we applied different staked time windows, and in particular 3-year and 7-year time dummies. Results, available upon request, reveal to be very similar to our baseline estimates.

13 The non-linearity of logit models does not allow us to directly interpret the interaction effects as they would be in linear models. Ai and Norton (2003) point out that interaction effects may have different signs for different levels of explanatory variables and related coefficients may not necessarily indicate the direction and magnitude of the effects.

14 Results of the estimated probabilities used to construct the graphs reported in Figs. 5, 6, and 7 are available upon request.

15 The coefficient of the interaction between the university dummy and time-period dummies in 1996–2000 is higher than in 1978–1985 (chi2(1) = 4.8) and lower than in 2006–2010 (chi(2) = 68.5).
